# Influence of Donor Tissue Factors on Detachment Rate in DSAEK Patients

**DOI:** 10.5402/2011/831051

**Published:** 2011-10-25

**Authors:** Daniel Demsey, Stephanie Baxter

**Affiliations:** Department of Ophthalmology, Hotel Dieu Hospital, Queen's University, 166 Brock Street, Kingston, ON, Canada K7L 5G2

## Abstract

*Purpose*. To determine whether the rate of graft detachment in patients undergoing the DSAEK procedure is influenced by the time elapsed between donor death and tissue implantation. *Methods*. Data on procedure outcome and donor tissue for patients undergoing the DSAEK procedure were reviewed. Data on time elapsed between harvest, processing, storage, and implantation of the tissue, as well as donor tissue endothelial cell count were obtained from reports made available from the Eye Bank of Canada. The adverse outcome of interest was graft detachment. *Results*. 71 cases were reviewed, with 14% resulting in detachment. The following time periods were compared between detachment and nondetachment groups: donor death to enucleation; enucleation to processing; duration of storage at the Eye Bank to implantation. No statistically significant differences were found (Student's *t*-test, *P* > 0.05). Endothelial cell counts of donor tissue were compared between the two groups, and no statistically significant difference was found (Student's *t*-test, *P* > 0.05). *Conclusion*. The range of processing times and endothelial cell counts in donor tissue available from the Eye Bank did not predict a change in the rate of graft dislocation in one surgeon's practice.

## 1. Introduction

The Descemet's stripping automated endothelial keratoplasty (DSAEK) procedure is primarily used to treat corneal endothelial dystrophies [1], and more recently to treat endothelial failure in previous penetrating keratoplasty [2]. Descemet's membrane and endothelium are removed through a small incision and replaced with donor tissue, which adheres to the recipient's inner corneal stroma [1]. The advantages of the DSAEK procedure when compared with the traditional penetrating keratoplasty (PK) technique have been previously described in the literature [1, 3–8]. The major postoperative complication of the DSAEK procedure is dislocation of the donor tissue from the corneal transplant bed, which has been reported to happen in 4–27% of patients [1, 5, 7, 9, 10]. Donor detachments are most likely attributed to surgeon error or a lack of donor tissue viability.

In Ontario, procured donor tissue is sent to the Eye Bank of Canada before being shipped to the transplanting surgeons for implantation. A period of time, typically hours, passes between the death of the donor and enucleation of the eye, and between enucleation and processing. The tissue then spends a period of days in storage at the Eye Bank before implantation in the recipient eye. Currently all DSAEK donor tissue is prepared by individual surgeons in Ontario at the site and time of transplantation, as precut DSAEK tissue is not available.

It is our hypothesis that a greater period of time spent in storage prior to processing results in a greater loss of tissue viability and concordantly leads to a higher rate of detachment. Additionally, it is hypothesized that a higher endothelial cell count indicates better tissue function and results in a lower detachment rate. The primary aim of our investigation is to determine whether these factors play a predictive role in tissue detachment in one surgeon's practice.

## 2. Methods

Data on donor tissue and procedure outcome were collected through a retrospective chart review of all patients in one academic surgeon's practice at Hotel Dieu Hospital in Kingston, Ontario. The investigation included all of the senior author's DSAEK patients from April 1, 2007 until March 30, 2010. Ethics approval was obtained through the Department of Health Sciences Ethics Review Committee.

All procedures were performed by one surgeon, the senior author. For all procedures the surgeon cut the donor tissue minutes prior to implantation in the patient. All tissue was cut using a Moria microkeratome, with a 300 micron head. No major problems occurred in preparing the tissue that might have contributed to DSAEK button detachment. All procedures were performed similarly, implanting the donor tissue using Goosey folding forceps. Four venting incisions were used in all patients, and interface fluid was drained via these incisions in all patients until no further fluid could be drained. All patients had a complete fill of air in their anterior chambers for 15 minutes after the drainage of fluid from the venting incisions, and about 50% of the air was removed just prior to concluding each case, to ensure no papillary block developed. All patients were kept in the recovery area, lying flat for 1 hour postoperatively. All patients were routine cases undergoing DSAEK for Fuchs' Endothelial Dystrophy or pseudophakic bullous keratopathy. None had complex anterior segments for any reason.

Data on length of time between donor death and tissue procurement, procurement and processing, and time spent in storage were taken from the Eye Bank reports that accompany the donor tissue. Preoperative endothelial cell count of the donor tissue was also taken from this document. Clinic reports on followup were reviewed and any evidence of tissue detachment was recorded.

## 3. Results

A total of 71 procedures were performed during the period of study, with 10 resulting in postoperative tissue detachment. This corresponds to a detachment rate of 14%.

Duration of time spent between enucleation, processing, and implantation, as well as endothelial cell count for the detachment and nondetachment groups are summarized in Table 1.

The time spent in each phase of processing was compared between the detachment and nondetachment group using a paired Student's *t*-test. This showed no statistically significant difference between the two groups, with the *P* values reported in Table 1.

Endothelial cell counts of the two groups were similarly compared using a paired Student's *t*-test, again showing no statistically significant difference between the 2 groups.

## 4. Discussion

As demonstrated by our results, there was no statistically significant difference in the storage and processing times between the detachment and nondetachment groups. This suggests that slight variations in donor tissue processing and storage times do not result in a higher incidence of DSAEK graft dislocation. This of course would apply only to this surgeon's practice, but may warrant further investigation. Also, it would only include intraoperatively prepared donor tissue and not precut tissue, prepared by the Eye Bank. However, Dapena et al. described success with a range of ages of precut tissue, suggesting that this result may apply to precut tissue as well [9]. They did not, however, make a direct comparison between a population of successful implantations and graft failures. Additionally, the use of surgeon-cut tissue has not been shown to have a different incidence of graft dislocation as compared to precut tissue [11, 12].

Endothelial cell count was also found not to be significantly different between the two groups, within the population of donor tissue available from the Eye Bank. Contrary to our initial expectation, the average cell count was actually slightly higher in the detachment group than in the nondetachment group, though this did not reach statistical significance.

These findings must be considered in light of the limitations of our study. The main limitation to this study is its limited scope, including only the practice of one surgeon. Perhaps a greater sample size could have shown a statistically significant difference. Additionally, the lack of precut tissue in our evaluation must be considered.

Neither length of processing time nor endothelial cell count appears to be significant predictors of graft dislocation in DSAEK procedures in one surgeon's practice. This suggests that in Kingston, all donor tissue from the Eye Bank being cut by the surgeon at the time of surgery has an equal chance of being successfully implanted, with the determinant of procedural success lying in the technique of the attending surgeon. A future direction for study could include various surgeons looking at their dislocation rates to see if this is the case in general, or that it was an isolated finding in one surgeon's practice.

## Figures and Tables

**Table 1:** Processing time and endothelial cell count.

	No detachment	SD	Detachment	SD	*P*
*N*	61		10		
Time to procurement (hours)	6.2	3.5	7.4	4.0	0.36
Time to processing (hours)	13.1	5.9	13.5	6.3	0.87
Time in bank (days)	3.9	2.2	4.6	2.2	0.36
Cell count	2647.4	244.5	2776.5	299.5	0.14
